# Ulnar Nerve Injury and Froment’s Test: A Case Report

**DOI:** 10.7759/cureus.6335

**Published:** 2019-12-10

**Authors:** David Cloete, Sa'ad Lahri

**Affiliations:** 1 Emergency Medicine, University of Stellenbosch, Cape Town, ZAF; 2 Emergency Medicine, Tygerberg Hospital, Cape Town, ZAF

**Keywords:** froment's test, froment's test, gunshot forearm, gunshot forearm, ulnar nerve injury, ulnar claw, upper limb injury, extremity injury

## Abstract

Traumatic, peripheral nerve injuries can be easily missed in the emergency department. The attending physician needs to maintain a high index of suspicion when reviewing patients with extremity injuries.

We present a case of a stable, 28-year-old male sustaining penetrating trauma to his right forearm with resultant, isolated ulnar nerve transection. Clinical findings and related anatomy are discussed pertaining to this patient's injury, with specific reference to Froment's test. This is a useful clinical adjunct when reviewing potential ulnar nerve injuries, demonstrating disruption of specific motor innervation to the thumb when such pathology exists. As a result, compensatory hyperflexion occurs with attempted thumb adduction, due to intact median nerve innervation of flexor pollicis longus. Early recognition of this pathology, whether isolated or concomitant, allows for early appropriate referral and improved patient outcomes.

## Introduction

South Africa (SA) ranks amongst the highest in terms of trauma and violence globally, with approximately 66 trauma presentations per 1,000 population [1]. Cape Town is one of the most violent cities in the world [2,3]. Approximately one-third of admissions to emergency centers in SA are due to injuries, a staggering figure when compared to first-world nations like the US (12%) and the UK (8%) [4].

Interpersonal violence continues to be a major contributor in this regard, particularly in the form of penetrating injuries. One study in a major center in SA noted 44.3% of patients sustaining fatal penetrating injuries, with ~37% and ~61% sustaining gunshot and stab wounds, respectively [5]. A recent, large, prospective registry-based study at a tertiary hospital in Cape Town noted that 10% of upper-limb gunshot injuries had associated nerve injury [6].

As emergency physicians working on the 'front line', it is imperative that we evaluate all trauma patients with a coordinated, step-by-step approach. In terms of peripheral extremity injuries identified during the initial trauma survey, these must always be evaluated for possible skeletal, vascular, and neurological compromise. Timely recognition of such injuries and early, appropriate referrals have a positive impact on patient outcomes, particularly in preventing long-term disability [6,7].

## Case presentation

**Figure 1:**
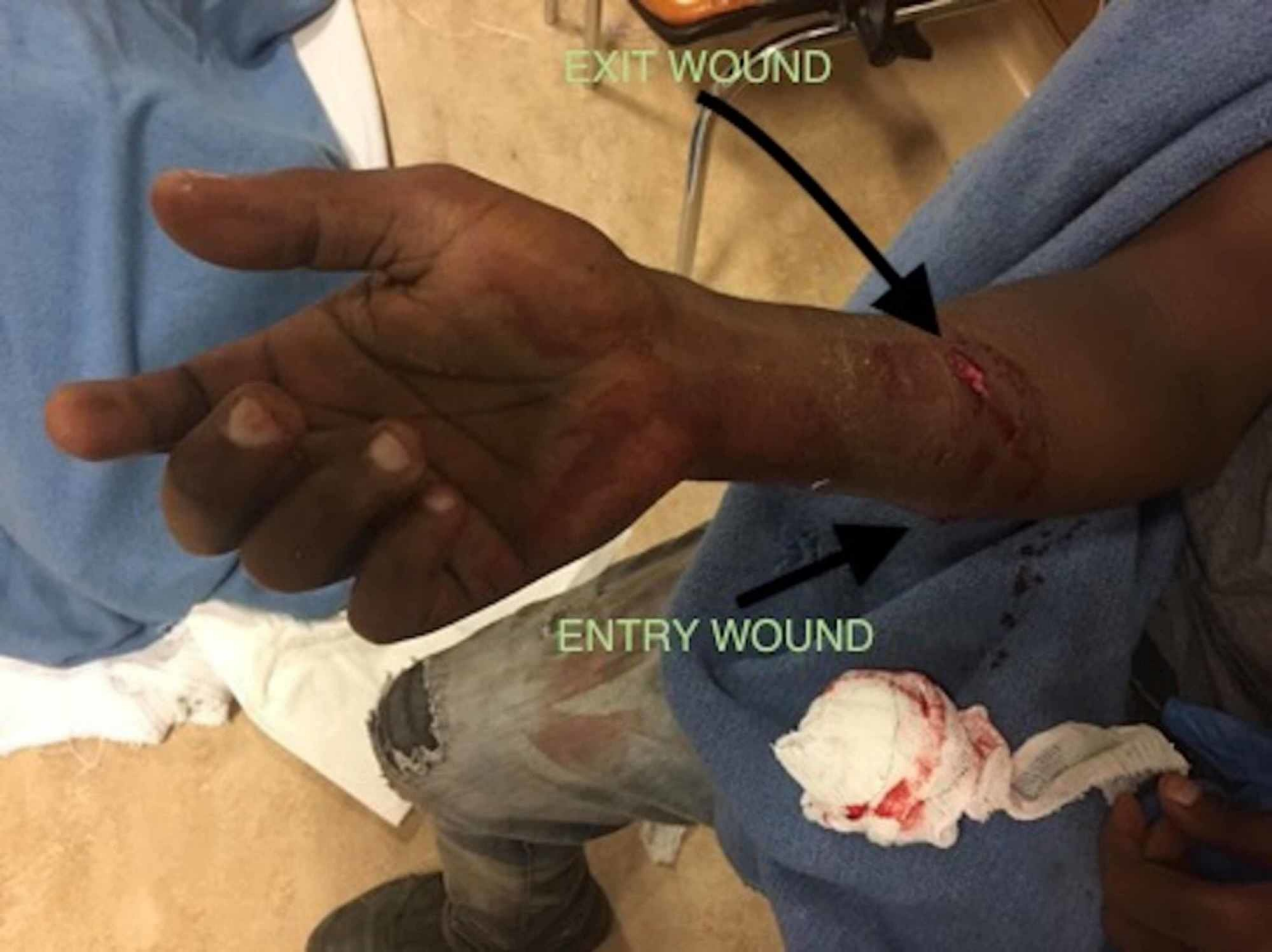
Gunshot wound on the right forearm

A 28-year-old male patient presented to our emergency department after sustaining a gunshot wound to the right forearm. The entry wound was located on the ulnar-dorsal aspect of his right mid-forearm. The exit wound was located on the volar aspect of the forearm and 3 cm distally to the elbow (Figure 1). Examination revealed absent sensation on his right 5th and ulnar half of the 4th fingers. The metacarpophalangeal joints of his 4th and 5th fingers were extended and the interphalangeal joints of the same fingers were flexed, suggestive of a ‘claw’. Froment’s test was positive.

The test was performed by asking the patient to hold a piece of paper in his hands, pinched between his thumbs and index fingers. The examiner then attempted to pull the paper away from the patient. It was noted that in order for the patient to maintain his hold on the affected side, his thumb flexed at the interphalangeal joint. The left, uninjured side demonstrated normal thumb adduction (Video 1).

**Video 1:** Performing Froment’s test to determine ulnar nerve injury

Peripheral pulses were noted to be equal bilaterally, and there were no other injuries. X-rays of the forearm revealed no fractures. Neurotmesis of the right ulnar nerve was suspected and he was referred urgently to the orthopedic surgery department for further care. The patient was taken to theatre for repair the same day.

## Discussion

The ulnar nerve is a mixed sensory and motor nerve, originating from the larger, terminal branch of the medial cord, receiving fibers from C8 and T1 nerve roots of the brachial plexus. Its superficial anatomical course makes it vulnerable to injury [8].

**Figure 2:**
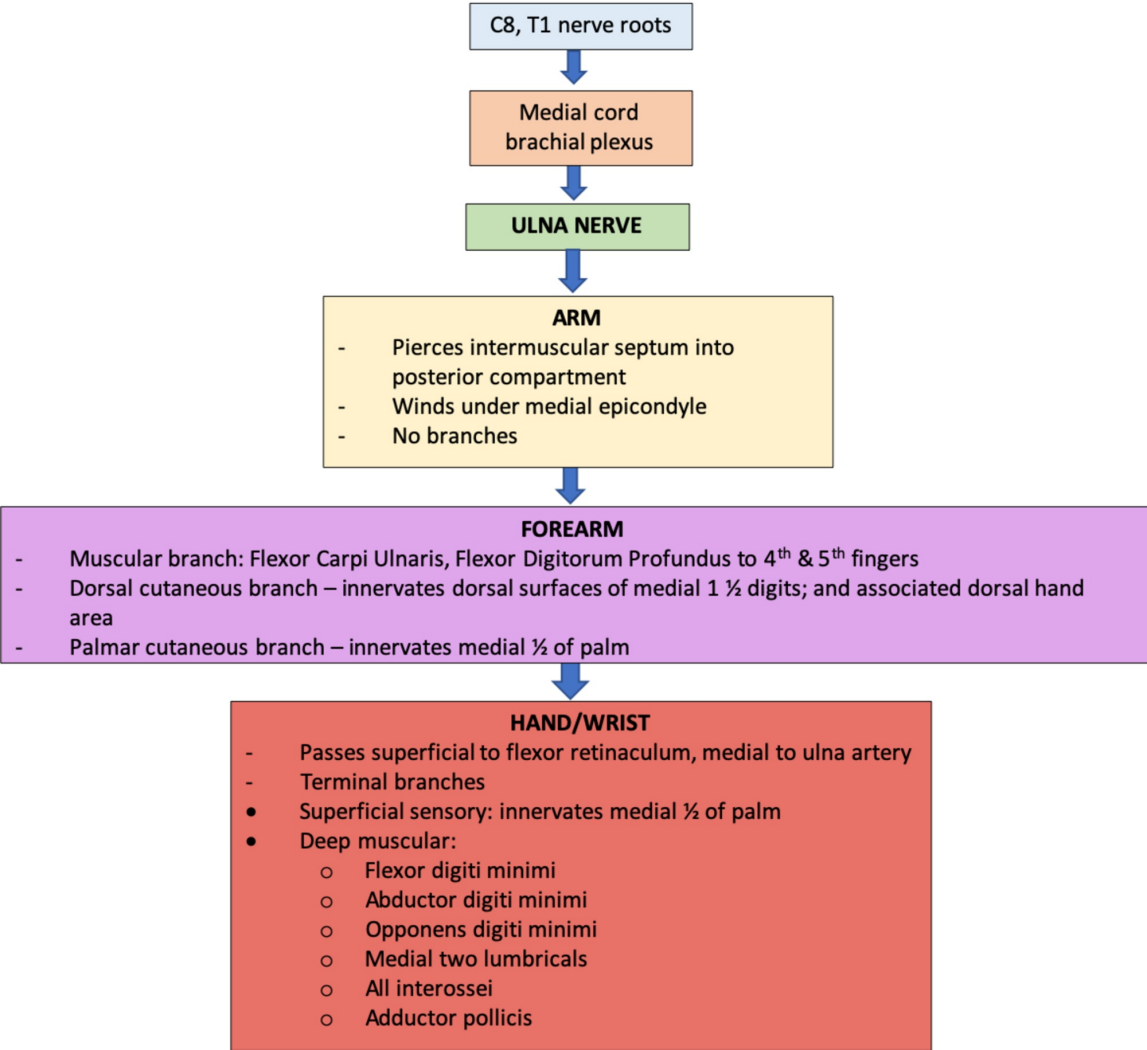
Course and innervation of the ulnar nerve

The ulnar nerve runs on the coracobrachialis muscle to the mid-arm, and thereafter pierces the medial intermuscular septum to enter the posterior compartment. It winds under the medial epicondyle and passes between the two heads of flexor carpi ulnaris to enter the forearm, supplying flexor carpi ulnaris and half of flexor digitorum profundus. In the lower forearm, dorsal and palmar cutaneous branches are given off. Finally, the ulnar nerve passes superficially to the flexor retinaculum and divides into two terminal branches: a superficial sensory branch supplying digital nerves to the skin of the little and medial half of the ring finger; and a deep motor branch supplying the hypothenar muscles, two lumbricals, the interossei and adductor pollicis (Figure 2) [8].

Clinical findings of upper limb injury, associated with ulnar nerve involvement, will depend on the location and the nature of the injury and may demonstrate motor or sensory deficits, or both as in this case [8,9]. Potential sensory involvement includes deficit to the palmar and dorsal surfaces of the hand, 5th finger and ulnar half of the 4th finger - the gunshot trajectory in this patient evidently damaged the ulnar nerve proximal to the dorsal and palmar cutaneous branches. Motor involvement was also noted in this case, and the patient's resting-hand position demonstrated a deformity known as an ulnar claw. This is due to the loss of interossei and lumbrical function of the 4th and 5th fingers with the result that the metacarpophalangeal joints hyperextend and the interphalangeal joints flex, combined with the unopposed action of the extensors and flexor digitorum profundus [8,10].

In addition, the ulnar nerve paradox was also observed, where less clawing is present with a higher ulnar nerve lesion in comparison to a lower lesion. This is explained by the inclusion of the ulnar half of flexor digitorum profundus in such proximal lesions, with the associated denervation resulting in relative relaxation of the interphalangeal joints [8-10].

Froment's test, the sensitivity or specificity for which has never been reported [11], is an adjunctive test that evaluates the integrity of the ulnar nerve. More specifically, it evaluates the innervation of the adductor pollicis muscle and interossei muscles, which adduct the thumb and extend the interphalangeal joint, respectively. With ulnar nerve disruption, the patient is unable to demonstrate a normal pinch grip, and the flexor pollicis longus muscle (itself innervated by the median nerve) will substitute for adductor pollicis, resulting in compensatory hyperflexion of the thumb [9,12-14].

## Conclusions

A nerve injury has the potential to drastically impact a patient's daily life, and it needs to be evaluated thoroughly when patients present with any extremity injury. This is particularly important in regions of the world faced with a high trauma burden where these potentially debilitating pathologies may present more subtly and exist concomitantly. A clear understanding of the basic anatomy allows the attending physician to consider potential nerve injuries and employ additional clinical tests to reinforce this suspicion. Froment's test, which helps to detect for disruption of the ulnar nerve, is one such clinical test that is quick and easy to perform. 
